# Identification and management of a novel Danshen leaf anthracnose caused by *Colletotrichum karstii* in *Salvia miltiorrhiza* Bunge in China

**DOI:** 10.3389/fpls.2025.1526038

**Published:** 2025-02-04

**Authors:** Haoyue Ma, Liguo Huang, Lulu Guo, Shan Chen, Jiale Liu, Changyun Liu, Yanxia Dou, Xianchao Sun, Lin He, Guanhua Ma

**Affiliations:** ^1^ College of Plant Protection, Southwest University, Chongqing, China; ^2^ Dezhou Academy of Agricultural Sciences, Shandong, China; ^3^ Chongqing Academy of Agricultural Sciences, Chongqing, China

**Keywords:** *Salvia miltiorrhiza*, anthracnose, *Colletotrichum karstii*, pathogen identification, biological characteristics

## Abstract

Danshen (*Salvia miltiorrhiza* Bunge), a member of the genus *Salvia* within the *Lamiaceae* family, holds significant economic and medicinal value. Regrettably, the emergence of a novel leaf anthracnose in 2020 has significantly impacted its cultivation, leading to decreased yield and compromised quality. This newly identified pathogen was meticulously isolated from affected leaves, employing meticulous single conidia isolation techniques. Subsequent confirmation of pathogenicity was achieved through strict adherence of Koch’s postulates. To ensure precise identification, morphological characteristics were supplemented with tandem sequence analysis targeting the rDNA internal transcribed spacer (*ITS*), β-tubulin (*TUB*), and histone (*His3*) regions. Combining molecular biology techniques with morphological observation and Koch’s postulates, the pathogen was conclusively identified as *Colletotrichum karstii*. Further investigations focused on understanding the environmental factors influencing the mycelial growth and sporulation of the pathogen. The optimum temperature for the growth of *C.karstii* is 25°C, the suitable light conditions are 12h light/12h dark or 24h dark, and the suitable pH is 5 to 9. Utilizing BIOLOG phenotypic analysis technique, the metabolic utilization of carbon and nitrogen sources by the pathogen was assessed across different temperatures (20°C, 25°C, and 30°C). Results indicated the highest utilization rates at 25°C, particularly for arbutin and L-tryptophan. Lastly, the efficacy of 15 chemical fungicides and six botanical fungiticide against *C. karstii* was evaluated in vitro, revealing fluazinam as the most potent inhibitor against mycelial growth with *EC_50_* of 0.0725 mg/mL for mycelium and 0.0378 mg/mL for spore germination, respectively. The 1 % osthole emulsion in water was found to have the strongest inhibitory effect on the growth of mycelium, with an EC50 value of 4.8984 µg/mL. Spore germination was most strongly inhibited by the 80 % ethylicin EC, which had an EC50 value of 0.5541 µg/mL. This study represents the first documentation of *C. karstii* as a causative agent of anthrax in Danshen, underscoring the significance of these findings for agricultural management and disease control strategies.

## 1 Introduction

Danshen (*Salvia miltiorrhiza* Bunge), the underground part of the *Salvia* plant, holds significant economic and medicinal value (Cheng, 2006). In recent years, the utilization of Danshen as a natural product has notably increased in both the United States and Europe (Kum et al., 2021). Danshen finds wide application in treating various ailments, including cardiovascular, cerebrovascular, and hyperlipidemia diseases (Qian et al., 2013). Clinically, Danshen is typically administered in compound preparations, such as the Compound Danshen Dripping Pill or Dantonic^®^ (T89) Capsule, which consists of Danshen and *Panax notoginseng* as primary ingredients. Notably, T89 stands as the world’s first compound Chinese herbal medicine to have successfully passed a multi-center phase 3 clinical trial regulated by the US Food and Drug Administration (FDA), currently undergoing FDA drug approval procedures (Xing et al., 2023a). Over recent decades, Danshen cultivation has seen extensive growth in Shandong, Sichuan, Henan, and Shaanxi provinces in China, with prospects for cultivation as an alternative crop in Mississippi, USA (Xing et al., 2023b). However, wild resources have dwindled while industrial consumption has surged (Zhang et al., 2024).

With the continuous expansion of the cultivation area of Danshen, the prevalence of diseases affecting this plant has escalated (Sa et al., 2022). Notably, root rot emerges as one of the most severe afflictions (Sa et al., 2022). Pathogens linked to Danshen root rot encompass *Fusarium solani*, *F. equiseti*, *F. oxysporum*, *F. proliferatum*, and *Alternaria tenuissima*, among others (Sa et al., 2022; Jin et al., 2020; Han et al., 2023; Song et al., 2024; Pu et al., 2022). Additionally, Danshen blight, instigated by *F. oxysporum* and *Alternaria* sp., presents a lesser concern (Yang et al., 2013). Furthermore, nematode diseases affecting Danshen, induced by *Meloidogyne incognita*, *M. arenaria*, and *M. javanica* (Pan et al., 2022; Wen et al., 2022), alongside Danshen leaf spot, attributed to *A. zinniae* and *Corynespora cassiicola* (Lu et al., 2018), have been documented. Additionally, Danshen mosaic disease, incited by cucumber mosaic virus (CMV), has been identified (Wu et al., 2008). However, the incidence of Danshen leaf anthracnose and its causative pathogen remain unreported.

Anthrax, caused by fungi belonging to the genus *Colletotrichum*, is a global plant disease. According to the IMI Descriptions of Fungi and Bacteria, *Colletotrichum* is classified under Ascomycota and Pezizomycotina, Sordariomycetes, Hypocreomycetidae, Glomerellales, Glomerellaceae, *Colletotrichum* (Talhinhas and Baroncelli, 2023). This fungus exhibits strong adaptability to hosts and can infect a diverse range of plants, including both monocots and dicots (Talhinhas and Baroncelli, 2023; Boufleur et al., 2021). Typically, it targets various plant parts such as roots, stems, leaves, flowers, fruits, and seedlings, inducing symptoms such as sunken necrosis on stems, flowers, and fruits, lesions on leaves, and even plant death, resulting in significant economic losses (Dean et al., 2012). Its taxonomic classification has evolved over more than a century, with numerous revisions, particularly with recent advancements in DNA technology. Currently, over 100 species of *Colletotrichum* have been described (Cannon et al., 2012). Due to its widespread distribution, destructive potential, and scientific significance as a model pathogenic system, *Colletotrichum* ranks among the ten most important fungal plant pathogens (Dean et al., 2012). *Colletotrichum karstii* is considered part of the *C. boninense* species complex (Damm et al., 2013). Initially reported to cause *Orchidaceae* anthracnose in China in 2011 (Youlian et al., 2011), this species has since been identified as the causal agent of anthracnose in various locations including the United States, Brazil, Spain, and Mexico (Mayorquin et al., 2019; Garcia-Lopez et al., 2020; Fernández-Herrera et al., 2020; Nascimento et al., 2019). However, there are no documented reports on its infestation of Danshen.

In this study, the occurrence of anthracnose in Danshen was documented and *C. karstii* was identified as the causal pathogen. Furthermore, an analysis of the fundamental biological characteristics of *C. karstii* was conducted and fungicides capable of effectively inhibiting its growth were screened. These findings offer a theoretical foundation for the scientific prevention and management of anthracnose in Danshen.

## 2 Materials and methods

### 2.1 Sample collection and fungus isolations

On June 3, 2020, suspected anthracnose symptoms were observed on Danshen leaves within a plantation located in Liangba Village, Hechuan District, Chongqing, China (29.939940°N,106.190213°E). To ascertain the causative pathogen responsible for these symptoms, ten strains of Danshen were acquired for pathogen isolation purposes. Tissue blocks measuring 4 mm×4 mm were excised from the interface between diseased and healthy areas on the leaves of Danshen. These tissue blocks were subsequently immersed in 75% alcohol for 30 seconds, followed by a 2-minute immersion in 5% NaClO solution. They were then rinsed thrice with sterile water and air-dried. The surface-sterilized tissue blocks were placed onto water agar (WA) plates and incubated under dark conditions at 25°C for three days. The outermost hyphal growth was carefully excised and transferred onto potato dextrose agar (PDA) supplemented with penicillin for further cultivation. After seven days of incubation, a small section of mycelium was inoculated into 100 mL of complete culture medium (CM) and cultivated at 28°C with agitation at 180 rpm to obtain a conidial suspension. The conidial suspension was appropriately diluted with sterile water and evenly spread onto WA plates. 36 single-conidia isolates were selected and transferred onto PDA medium for successive subcultures to achieve purified strains. Detailed compositions of the culture media are provided in **Supplementary Table S1**.

### 2.2 Identification of the isolations

Firstly, molecular identification was conducted by extracting DNA from the purified 36 single-conidia isolates following the protocol outlined in the HiPure Fungal DNA Mini Kit (Guangzhou Magen Biotech Co., Ltd., D3171-02, Guangzhou, China). The intra ribosomal transcribed spacer (*ITS*) region of the ribosomal rDNA-16S spacer sequence was amplified using primer pairs ITS1/ITS4 as described (Schoch et al., 2012). Based on the obtained results, these isolates were categorized into three distinct strains denoted as DSL, DSN, and DSM. Subsequent analysis involved the DSL strains, which were further examined using primer pairs for the *ITS*, β-tubulin gene (*TUB*) (primers Bt2a/Bt2b), and histone (*His3*) genes; while the DSN strains were analyzed using primer pairs for *ITS*, large subunit rRNA (*LSU*) (primers LROR/LR7), and the second largest RNA polymerase subunit (*RPB2*) (primers RPB2-5F2/RPB2-7cR) (Glass and Donaldson, 1995). For the DSM strains, amplification was performed targeting the elongation factor 1-α region (*TEF1*) (primers EF1-728F/EF1-986R), allergen gene (*Alt al*) (primers Alt-R/Alt-F), and *RPB* (Hong et al., 2005). See **Supplementary Table S2** for detailed primers. The total PCR reaction system comprised 20 μL, including 10 μL 2 × Es Taq MasterMix (Jiangsu ComWin Biotech Co.,Ltd., CW0690H, Jiangsu, China), 1 μL each of forward and reverse primers(10μmol/L), 1 μL DNA template, and 7 μL ddH_2_O. PCR amplification conditions were set as follows: initial denaturation at 95°C for 5 min, denaturation at 95°C for 30 s, followed by 32 cycles of annealing at the corresponding temperature for 30 s (**Supplementary Table S2** for annealing temperatures), extension at 72°C for 60 s, and a final elongation step for 10 minutes. Amplified products were subjected to agarose gel electrophoresis for detection and subsequently sent to Sangon Biotech (Sangon, Shanghai, China) for sequencing. The sequence fragments obtained after sequencing were assembled using DNAMAN software. Subsequently, the assembled sequences were subjected to homology comparison in the NCBI GenBank database. Upon completion of the alignment, the related sequences of various type species (ex-type) with high homology (ranging from 99% to 100%) in the database were downloaded for alignment and cluster analysis (**Supplementary Table S3**). PhyloSuite software was employed to compare and trim the base sequences of the reference strain and the test strain. The processed sequences were then analyzed in Mega 7.0, where the maximum likelihood method was used to construct the ML tree, and the bootstrap support analysis was set to 1000 replicates.

For morphological identification, the isolated and purified strain DSL underwent cultivation in darkness at 25°C for a duration of 5 days. Following this incubation period, the colony diameter was assessed using a cross-method to examine color, texture, density, and colony margin characteristics. Conidia and appressoria were induced utilizing the methodology outlined by Wang and Fung (Fang et al., 2018; Wang et al., 2017). Microscopic observations were conducted using the BK series biological microscope (Nikon Precision Machine (Shanghai) Co., Ltd., Eclipse E200, Shanghai, China). Conidia, sporulation structures, setae, ascospores, appressoria of conidia, and mycelia were meticulously examined and photographed, with measurements taken of ascospores, conidia, and appressoria sizes (n≥50).

### 2.3 Pathogenicity test of the isolations

Based on the molecular identification results, the pathogenicity of three different strains was assessed. One representative strain was chosen from each group, designated as DSM01, DSL01, and DSN01, respectively. The pathogenicity testing involved inoculating mycelium blocks onto healthy and disinfected Danshen leaves using 75% alcohol. Micropores (5 to 6 in number) were created within a 5 mm diameter area on the leaf surface. A 5 mm diameter agar block colonized with the respective strain was inverted onto the prepared leaf, while a PDA culture medium block of the same size served as a blank control (CK). Four inoculated pieces were placed on each tray, with a total of nine plates. Following incubation in darkness at 25°C for 2 days, disease symptoms were observed and recorded. Subsequently, reisolation of the pathogen from the inoculated leaves was performed to fulfill Koch’s postulates (Than et al., 2008). The pathogenicity of DSL01 was further evaluated through spore suspension inoculation. DSL01 was cultured on PDA medium at 25°C in darkness for 30 days. A conidial suspension mother solution was prepared using sterile water, and the spore concentration was adjusted to 10^5^ spores/mL. The spore suspension was evenly sprayed onto the leaves of annual Danshen plants, and the plants were enclosed in plastic bags within a greenhouse set at 25°C. Blank controls consisted of Danshen plants sprayed with sterile water. After 48 hours, the plastic bags were removed, and the plants were continuously cultivated in the greenhouse. Disease occurrence was monitored every 24 hours, and symptoms were observed, recorded, and photographed accordingly.

### 2.4 Growth characteristics of strain DSL

Next, an analysis of the growth characteristics of strain DSL01 was conducted, with an examination of its growth and sporulation traits under varying temperatures being initiated. A DSL cake, 5 mm in diameter, was obtained from the periphery of the colony and inoculated at the center of a PDA plate. Subsequent incubation was carried out in darkness across 10 temperature gradients: 5°C, 10°C, 15°C, 20°C, 25°C, 30°C, 32°C, 33°C, 35°C, and 40°C. Colony diameters were measured and recorded, with each treatment replicated thrice. For the sporulation characteristics at different temperatures, following a 30-day incubation period, conidial suspensions were prepared in each treatment dish by adding an equal volume of sterile water. Spore counts were conducted using a hemocytometer, and the spore quantity was calculated accordingly. Each treatment was replicated three times to ensure statistical robustness.

To examine the growth characteristics under varied lighting durations, a 5 mm diameter DSL cake was inoculated at the center of PDA plates. Three lighting conditions were established: 12 hours of light followed by 12 hours of darkness (12 h light/12 h dark), continuous 24-hour light (24 h light), and continuous 24-hour darkness (24 h dark). The light intensity was maintained at 1500 lux, and colony diameters were measured and recorded over a 7-day period at 25°C. After 30 days, spore production was quantified, with each treatment being replicated three times to ensure reliability and statistical significance.

To investigate the mycelial growth and sporulation characteristics under varying pH conditions, PDA was utilized as the base medium. The pH of the medium was adjusted to levels ranging from 4 to 11 using 0.1% HCl and 0.1% NaOH solutions, resulting in eight distinct pH gradients. DSL cakes, each with a diameter of 5 mm, were inoculated onto plates containing the respective pH-adjusted medium. Subsequent incubation was conducted at 25°C in darkness for a duration of 7 days, during which colony diameters were measured and recorded. Spore production was assessed after 30 days, with each treatment being replicated three times to ensure robustness and reliability of the results.

In final analysis, the impact of various temperatures on the pathogenicity of DSL was examined. DSLs were inoculated onto fresh leaves of Danshen using the mycelium inoculation method. The inoculated Danshen leaves were then cultivated under three distinct temperature gradients: 20°C, 25°C, and 30°C, with 10 replicates for each gradient. After a 7-day incubation period, the diameter of the lesions on the leaves was measured, and subsequently, the disease index was calculated.

For the analysis of these results, statistical analyses were conducted using SPSS software (version 23.0). One-way analysis of variance (ANOVA) was employed to compare multiple groups of data, followed by *post-hoc* LSD test for pairwise comparisons, with a significance level set at *p*< 0.05.

### 2.5 BIOLOG redox analysis of strain DSL

To conduct BIOLOG redox analysis, DSLs were initially inoculated onto PDA medium following the method outlined by Khatri’s method (Khatri et al., 2013). After a 30-day incubation period, FF-IF inoculum solution was introduced into the PDA medium to adjust its spore suspension until achieving a light transmittance of 62% T. Subsequently, 11.76 mL of the prepared spore suspension was transferred into a V-shaped groove, followed by the addition of 0.12 mL of BIOLOG Redox Dye Mix D and 0.12 mL of solution B. After thorough mixing, the spore suspension was promptly inoculated into a Petri dish. Next, 100 μL of the spore suspension was added to each well of the microwells in the PM1 plate using a multichannel pipette. For PM3B, 0.12 mL of solution A was added using the same reagents. The inoculated PM1, PM2A, and PM3B plates were swiftly placed into the BIOLOG system incubator and cultured at 20°C, 25°C, and 30°C for 7 days. The OmniLog 2.4 software was utilized to set reading parameters and collect data every 15 minutes, enabling observation of carbon and nitrogen source utilization for DSL. Subsequently, the Biolog OL_FM_1.2.exe software was employed to convert the carbon and nitrogen metabolism phenotype data of each strain, while the Biolog OL_PR_1.2.exe software was used for comparison. The peak area was chosen as the comparison parameter, where larger values indicate stronger metabolism of the detected carbon and nitrogen source materials by the strain (Jia et al., 2024). Finally, the utilization rates of different substances were plotted into a heat map to highlight highly utilized substances.

### 2.6 *In vitro* screening of fungicides against DSL

Chemical fungicides with eight distinct mechanisms(**Supplementary Table S4**) and botanical fungicides with five distinct mechanisms (**Supplementary Table S5**) of action were chosen to assess their inhibitory effects on DSL *in vitro*. In addition to 95% triadimefon original medicinal anhydrous ethanol solution dissolved, 97.3% carbendazim original medicinal 0.1% hydrochloric acid solution dissolved, each fungicide was formulated into a stock solution with a concentration of 1.0 × 10**^4^** μg/mL using DMSO. Six botanical fungicides was formulated into a stock solution with a concentration of 1.0 × 10**^3^** μg/mL using sterile water. Subsequently, the stock solutions were diluted to generate five concentration gradients.(**Supplementary Table S6**) and individually mixed with PDA culture medium. Each concentration gradient was replicated three times, with a treatment containing an equivalent volume of DMSO or sterile water serving as the CK. A 5 mm diameter mushroom cake was inserted into the center of each drug-containing plate, followed by incubation in darkness at 25°C for 7 days to determine the inhibition rate. For spore germination inhibition assays, a spore suspension with a concentration of 10**^5^** spores/mL was prepared. Subsequently, 500 μL of the spore suspension was placed on a concave glass slide, and five different concentration gradients of the test fungicides (**Supplementary Table S7**) were added. Each concentration was replicated three times, with sterile water added as a CK. Incubation was carried out at 25°C in darkness until the spore germination rate in the control group exceeded 95%. Following this, the spore germination rate in the treatment group was observed, recorded, and used to calculate the spore germination inhibition rate. The toxicity regression equation (y=ax+b), correlation coefficient (R), *EC50* value, and its 95% confidence interval were computed for each fungicide (Fan et al., 2022).


$$\text{percent inhibition}=\frac{\text{radial growth of the fungus in the control}-\text{radial growth of the fungus in the treatment}}{\text{radial growth of the fungus in the control}-5\text{mm}}\times 100$$

Inhibition efficiency of the fungicides


$$\text{precent inhibition}=\frac{\text{germination rate of fungal spores in the control group}-\text{germination rate of fungal spores in the treatment}}{\text{germination rate of fungal spores in the control group}}\times 100$$

The calculation formula of spore germination inhibition rate

## 3 Results

### 3.1 Field symptoms of leaf anthracnose in Danshen

**Figure 1:**
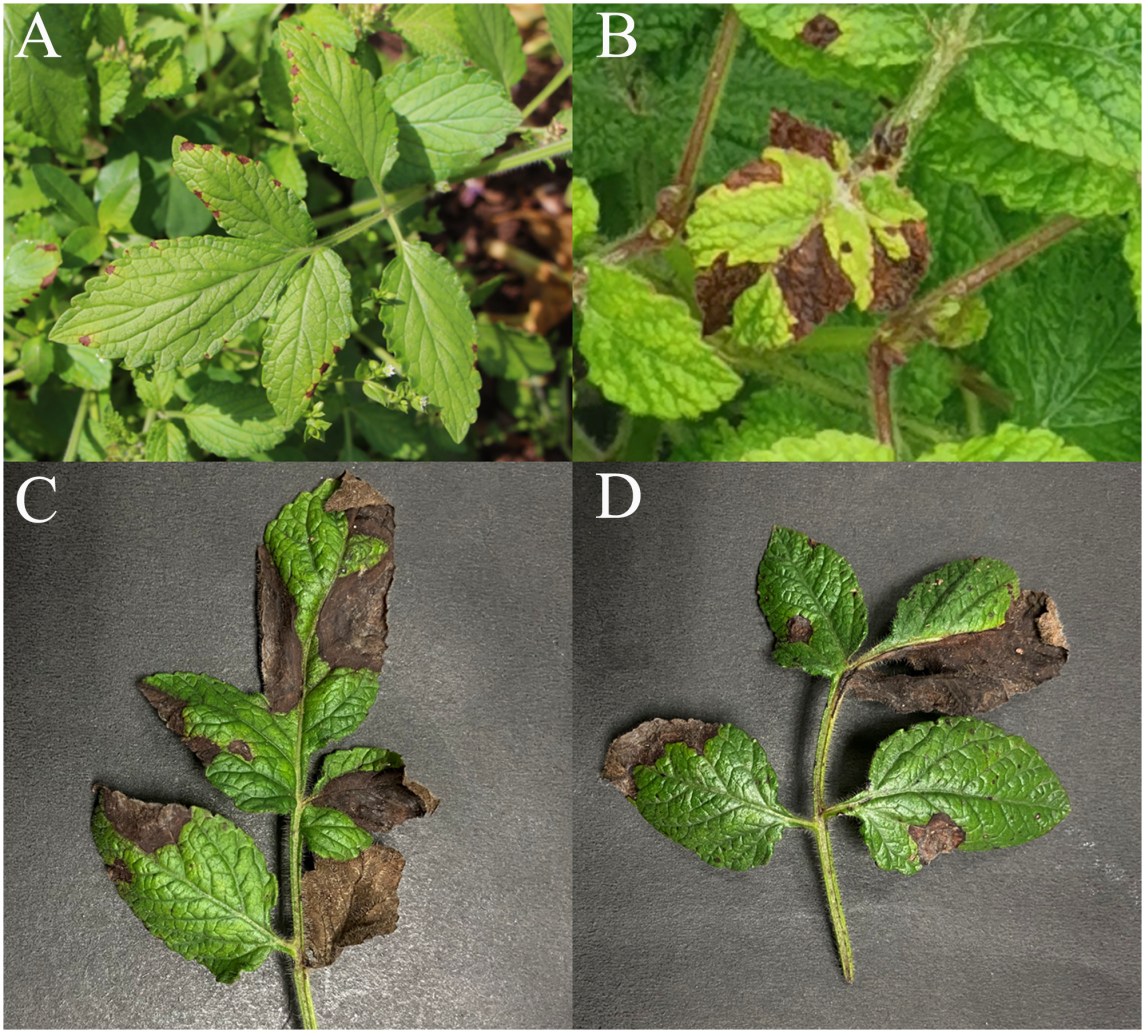
Field manifestations of anthracnose on Danshen leaves. **(A)** Initial manifestations of anthracnose on Danshen leaves; **(B)** Preliminary indications of anthracnose observed on Danshen leaves, **(C, D)** Later period of anthracnose symptoms observed on Danshen leaves.

In June 2020, a field survey of Chinese herbal medicine revealed widespread occurrences of leaf anthracnose in Danshen planting areas. Primarily affecting the leaves of Danshen, the disease initially manifested in the upper and middle leaves. Subsequent observation of field-wide symptoms revealed that during the early stages of the disease, light brown spots appeared on the leaf tips and edges (**Figure 1A**). As the disease progressed to the middle stage, the lesions expanded, transitioning from light brown to dark brown, with irregular shapes and distinct healthy boundaries (**Figure 1B**). In the advanced stages of the disease, the lesions merged, causing the leaves to become dry and brittle, ultimately leading to wilting and withering. Severe damage could result in the demise of the entire plant (**Figures 1C, D**).

### 3.2 *Colletotrichum karstii* is the causative agent of anthracnose on Danshen leaves

**Figure 2:**
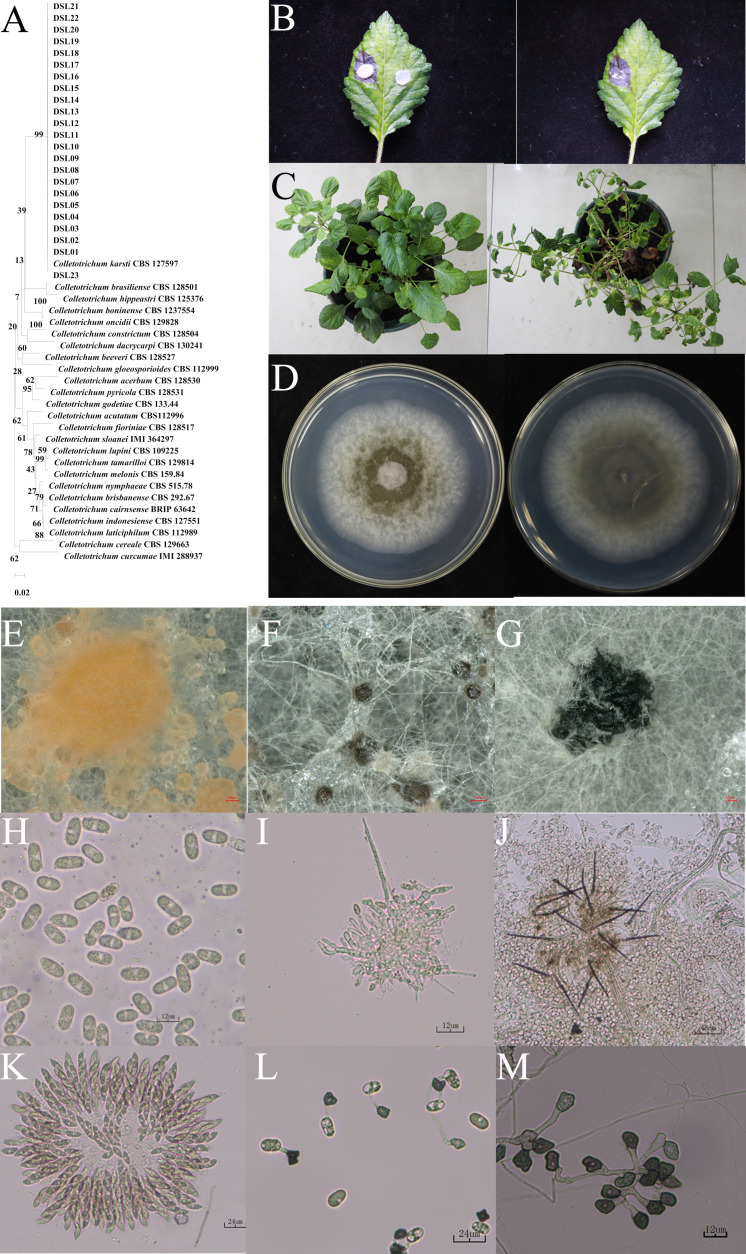
Identification of DSL as *Colletotrichum karstii*. **(A)** Phylogenetic tree depicting strains DSLs based on *ITS*, *TUB*, and *His 3* gene sequences. The maximum likelihood method with 1000 bootstrap iterations was employed, and MEGA X facilitated visualization. **(B)** Symptoms induced by strain DSL01 on the 7th day post-inoculation (dpi) of wounded Danshen leaves. **(C)** Symptoms on the 12th dpi with spore suspension inoculation of strain DSL01 on wounded Danshen leaves. **(D)** Colony morphology of strain DSL01 on PDA medium, showing top (left) and bottom (right) views after 5 days of culturing. **(E-M)** Microscopic observation of DSL01, revealing conidium piles, ascus, sclerotia, conidia, conidiophores, acervulus, ascospores, conidial appressoria, and hyphal appressoria. The scale bar for panels **(C-E)** is 100 µm, and for panels **(F-K)**, it is 12 µm.

To elucidate the pathogen responsible for anthracnose on Danshen leaves, 36 strains were isolated from 10 diseased leaves by employing a single spore isolation method. Initially, preliminary molecular identification was conducted for these 36 strains using the *ITS* region. The results revealed that the *ITS* sequences of these 36 strains were preliminarily classified into three fungal genera: *Colletotrichum* spp., *Alternaria* spp., and *Boeremia* spp., designated as DSL, DSN, and DSM, respectively. Subsequently, to further confirm their intraspecific classification, intragenus-specific primers were utilized to identify species within their respective genera. For DSL strains, *ITS*, *TUB*, and *His3* genes were employed; for DSN strains, *ITS*, *LSU*, and *RPB2* were utilized; and for DSM strains, primers targeting *TEF*, *Alt-al*, and *RPB*. Sequence numbers of the identified strains were then retrieved from literature and online databases, and the Maximum Likelihood (ML) method was used to construct a phylogenetic tree for the three types of pathogens using the aforementioned three genes. The results revealed that 23 DSL-type strains clustered together with *Colletotrichum karstii* with a bootstrap value of 99% (**Figure 2A**); 7 DSN-type strains clustered with *Alternaria alternata* (**Supplementary Figure S1**) with a bootstrap value of 97%; and six DSM-like strains clustered with *Boeremia exigua* (**Supplementary Figure S2**) with a bootstrap value of 100%. These results demonstrate that these three fungi may serve as causative agents of Danshen leaf anthracnose.

Subsequently, to accurately identify the pathogen responsible for Danshen leaf anthracnose, the pathogenicity of the three types of pathogens was assessed. Representative strains (DSL01, DSN01, and DSM01) were selected from each type, and their pathogenicity towards Danshen leaves was evaluated following Koch’s postulates. Five days post-wound inoculation with the DSL01 strain, brown lesions emerged on the leaves of Danshen, progressively expanding over time (**Figure 2B**). Conversely, leaves inoculated with the DSN01 and DSM01 strains did not exhibit any disease symptoms (**Supplementary Figures S3, S4**). Subsequently, it was verified whether anthracnose on Danshen leaves could be induced by the spore suspension of the DSL01 strain. Seven days post-inoculation, small brown spots appeared on the leaf tips and edges, rapidly expanding and leading to wilting and eventual leaf detachment by day 12 (**Figure 2C**). Both inoculation methods resulted in anthracnose on Danshen leaves, displaying dry and brittle lesions mirroring those observed in the original diseased samples. Re-isolation of lesions from Danshen leaves inoculated with the DSL01 strain yielded sequences consistent with DSL01 strain through ITS amplification. Subsequent pathogenicity testing of all 23 DSL strains corroborated these findings, strongly indicating DSL01 strain as the causative agent of anthracnose on Danshen leaves.

Subsequently, the representative strain DSL01 was focused on and a detailed observation of its morphological characteristics was conducted. It was revealed by the findings that a white and gray appearance was exhibited by the initial mycelium of the pathogenic fungus DSL01 which was cultivated on PDA medium, appearing fluffy with abundant aerial mycelium and regular margins. As the colony expanded, pigmentation began to develop internally, and the colony’s reverse side transitioned from colorless to yellowish-brown. After 7 days of incubation in the dark at 25°C, the colony effectively covered the plate, reaching a diameter of 90 mm (**Figure 2D**). Moreover, black sclerotia develop during the later stages of growth (**Figure 2G**). In the asexual state, the conidial masses appeared orange-yellow, while the conidial discs displayed a dark brown hue, often surrounded by numerous black or brown setae (**Figures 2E, J**). Conidiophores were observed to be colorless and short (**Figure 2I**). The conidia were straight-cylindrical with rounded ends, occurring singly, transparent, and containing two oil globules, with dimensions measuring 12.7 ± 1.1 × 5.7 ± 0.5 μm (**Figure 2H**). Both conidial and mycelial appressoria appeared as single cells, dark brown to black in color, irregular in shape, with dimensions of 7.8 ± 1.4 × 7.2 ± 1.3 μm and 10.2 ± 1.6 × 5.7 ± 1.5 μm, respectively (**Figures 2L, M**). In the sexual state, the ascostroma appeared black and spherical, while the asci exhibited a rod-shaped morphology with slightly pointed ends, containing 6 to 8 ascospores (**Figure 2F**). Ascospores were observed to be simple, colorless, and measured 14.8 ± 1.1 × 6.1 ± 0.6 μm (**Figure 2K**). These morphological characteristics were consistent with the reported traits of *C. karstii* (Mayorquin et al., 2019; Nascimento et al., 2019). Therefore, through comprehensive molecular and morphological identification, the DSL01 strain responsible for anthracnose on Danshen leaves was conclusively identified as *C. karstii*.

### 3.3 Growth characteristics of *C. karstii* strain DSL01 under different environments

**Figure 3:**
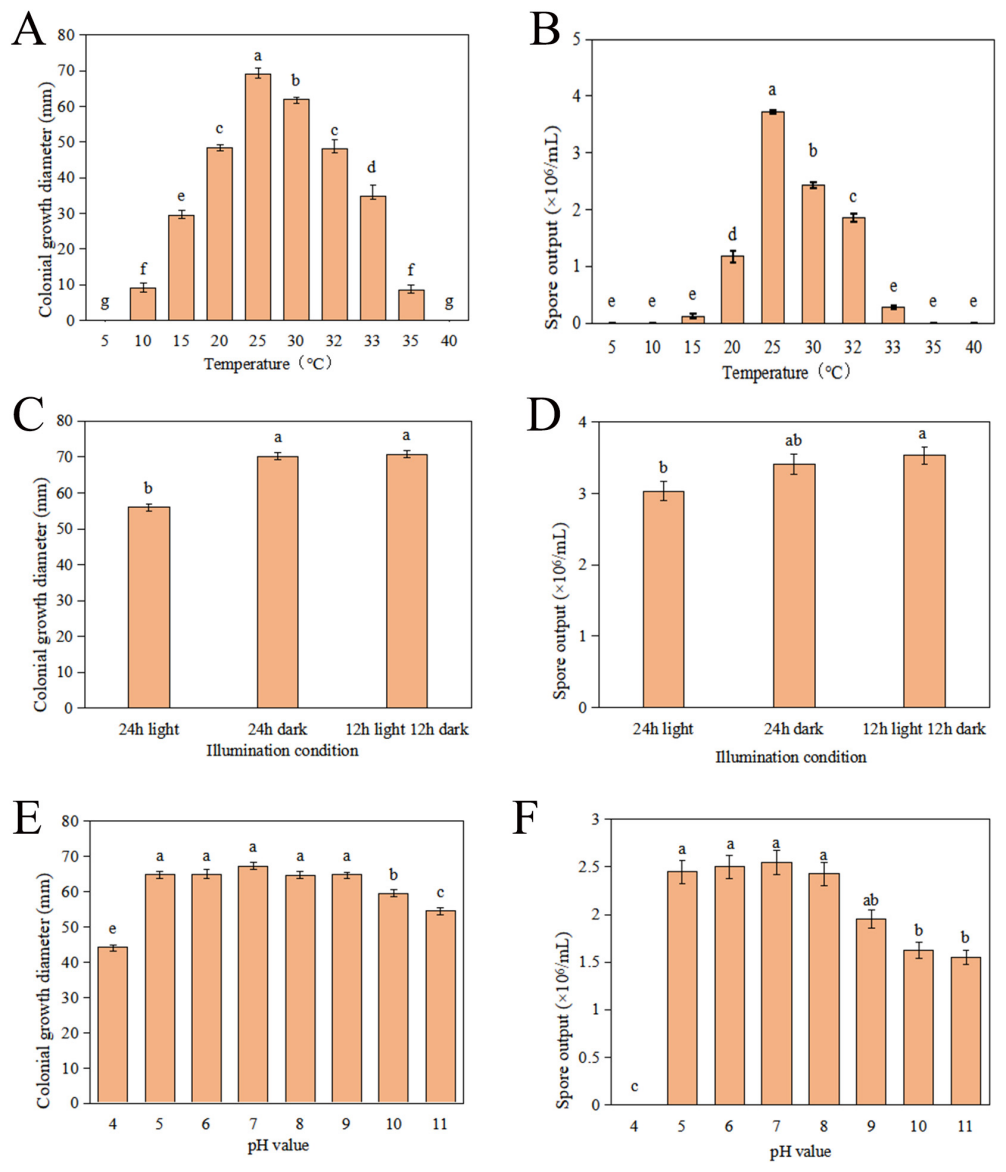
Growth characteristics of strain DSL01 under various environmental conditions. **(A)** Impact of different temperatures on strain DSL01 growth on PDA medium. **(B)** Influence of different temperatures on sporulation of strain DSL01. **(C)** Impact of different lighting conditions on strain DSL01 growth on PDA medium. **(D)** Influence of different lighting conditions on sporulation of strain DSL01. **(E)** Effect of varying pH levels on strain DSL01 growth on PDA medium. **(F)** Influence of varying pH levels on spore production of strain DSL01. All experiments had been repeated three times with similar results. Values represent means ± standard error (SE) from three biological replications. The statistical analyses were performed using AVONA test (LSD, *P*< 0.05). Lowercase letters indicate statistically significant differences between mean values (p < 0.05).

To comprehensively understand the growth characteristics of *C. karstii* strain DSL01 and devise effective strategies for the management of Danshen leaf anthracnose, an investigation into its mycelial growth and sporulation across varying temperatures was conducted. The findings revealed that strain DSL01 exhibited robust growth within a temperature range of 10 to 35°C, with growth ceasing at temperatures below 5°C and above 40°C. Mycelial diameter displayed a pattern of initial expansion followed by reduction. Optimal mycelial growth occurred at 25°C, with substantial sensitivity observed within the 30 to 35°C range (**Figure 3A**). Similarly, the effect of temperature on spore production demonstrated a trend of initial increase followed by decrease, peaking at 25°C, where spore production reached 3.73×10^6^ spores/mL. Notably, mycelial growth occurred at both 10°C and 35°C, albeit without spore production. Consequently, 25°C emerged as the optimum temperature for strain DSL01 growth, with maximal mycelial growth and sporulation observed at this temperature, highlighting its significance compared to other temperatures (*P*< 0.05) (**Figure 3B**). Concurrently, the pathogenicity of DSL01 at temperatures of 20°C, 25°C, and 30°C was assessed. It was revealed by our findings that the pathogenicity of the strain DSL01 was significantly influenced by temperature, with notable discrepancies observed across different temperature conditions. Specifically, DSL01 strain exhibited pathogenic activity on Danshen leaves within the temperature range of 20 to 30°C. At 20°C, the lesions exhibited the smallest diameter, indicative of weaker pathogenicity. Conversely, at 25°C, the lesions displayed the largest diameter, suggesting peak pathogenic activity, thus identifying 25°C as the optimal pathogenic temperature (**Table 1**). These investigations elucidated the fundamental pathogenic characteristics of *C. karstii* strain DSL01.

**Table 1:** Pathogenicity of strain DSL01 to Danshen leaves at different temperatures.

Temperature (°C)	Lesion diameter (mm)
20	9.75 ± 0.73 c
25	23.31 ± 1.76 a
30	15.44 ± 0.62 b

All experiments had been repeated three times with similar results. Values represent means ± standard error (SE) from three biological replications. The statistical analyses were performed using AVONA test (LSD, *P< 0.05*).

Simultaneously, the influence of different lighting conditions on DSL01 was investigated. The findings reveal that DSL01 strain exhibits growth across varying light regimes, including 24 hours of light, 24 hours of darkness, and a 12-hour light/12-hour darkness cycle. Mycelial growth thrived under both 24 hours of darkness and the 12-hour light/12-hour darkness cycle, with no significant difference observed between them. Conversely, mycelial growth was notably slower under continuous illumination for 24 hours (**Figure 3C**). Regarding spore production, the 12-hour light/12-hour dark cycle resulted in the highest spore yield, with 3.53×10^6^ spores/mL, followed closely by the 24-hour dark condition, yielding 3.41×10^6^ spores/mL. The difference between these conditions was not significant (**Figure 3D**). In summary, DSL01 strain exhibits robust growth under 12 hours of light, 12 hours of darkness, and continuous darkness for 24 hours, with no notable disparity between the latter two conditions.

Subsequently, the growth characteristics of strain DSL01 under various pH conditions were examined, and it was observed that growth of strain DSL01 was exhibited across a pH range from 5 to 9. The growth pattern displayed an initial increase followed by a subsequent decrease. Notably, within the pH range of 5 to 9, there was no significant disparity in hyphal growth, with optimal growth observed at pH 7, boasting an average diameter of 70.66 mm (**Figure 3E**). Regarding conidial production, strain DSL01 demonstrated the ability to produce conidia across pH values ranging from 5 to 11, except for pH 4. Among these, the highest spore yield was observed at pH 7, reaching 2.55 × 10^6^ spores/mL. However, there was no significant difference in spore production between pH values of 5, 6, 8, and 9 (**Figure 3F**). Overall, the optimal pH for the growth of the strain DSL01 ranged from 5 to 9, with a preference for neutral to alkaline environments. These growth characteristics delineate the fundamental biological attributes of strain DSL01, offering a theoretical foundation for enhanced prevention and management of Danshen leaf anthracnose.

### 3.4 Utilization of carbon, nitrogen, phosphorus and sulfur sources by *C. karstii* strain DSL01

**Figure 4:**
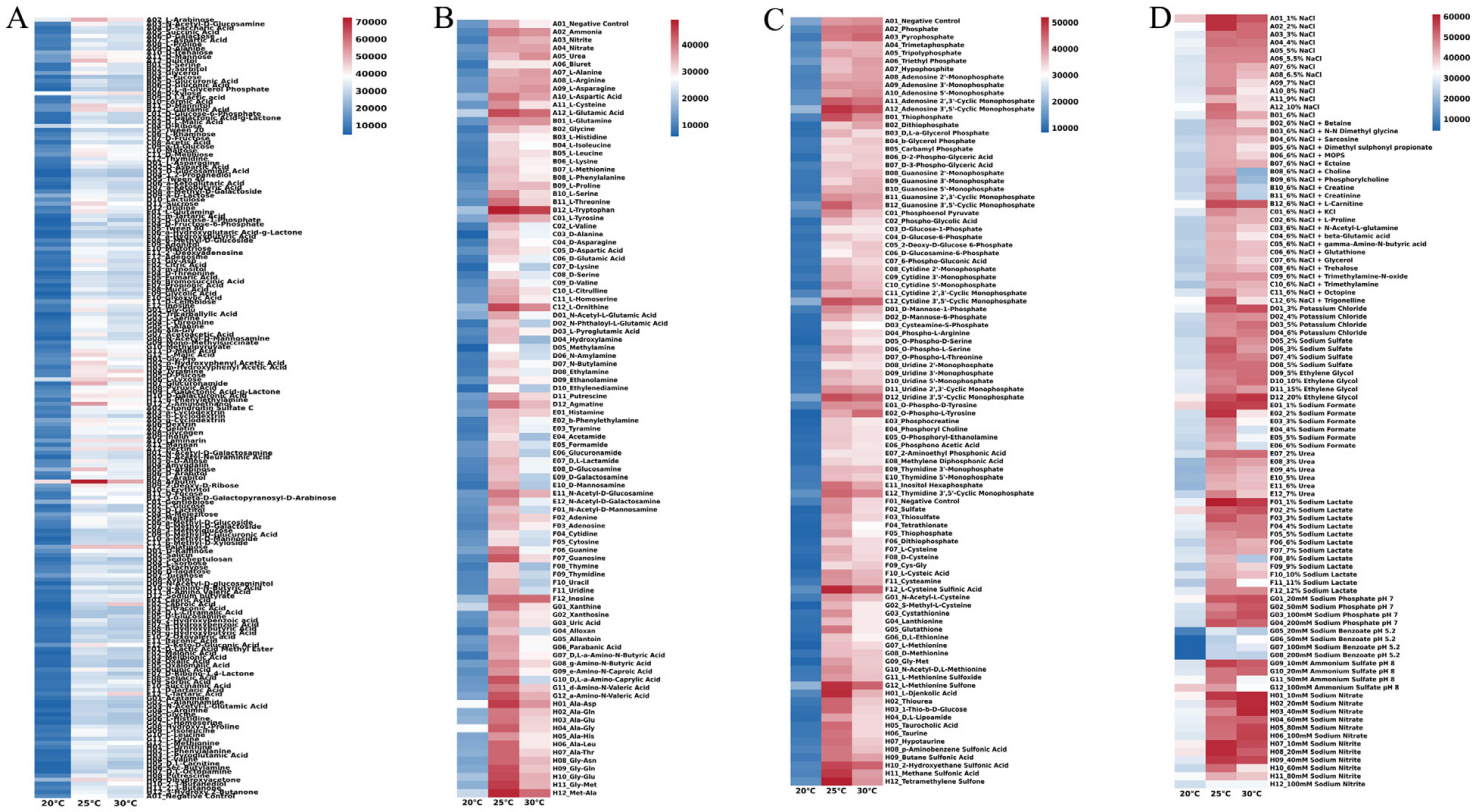
Metabolic utilization of various sources by strain DSL01 at different temperatures using BIOLOG redox system. **(A)** Utilization of 190 nitrogen sources by strain DSL01 at different temperatures. **(B)** Utilization of 95 carbon sources by strain DSL01 at different temperatures. **(C)** Utilization of 59 phosphorus sources and 35 sulfur sources by strain DSL01 at different temperatures. **(D)** Metabolic phenotype of strain DSL01 under varying temperatures, osmotic pressures, and ionic strengths.

Subsequent analyses involved examining the metabolic phenotype of strain DSL01 to elucidate its correlation with pathogenicity. Employing the BIOLOG redox system, the metabolic phenotype of strain DSL01 across different temperatures—20°C, 25°C, and 30°C—was scrutinized due to the significant pathogenicity observed at these temperatures. The findings revealed both commonalities and disparities in the utilization of metabolic carbon sources by DSL01 at varying temperatures. At 20°C, 25°C, and 30°C, the strain demonstrated the ability to metabolize 90, 98, and 95 carbon sources, respectively, with utilization rates of 47.37%, 51.58%, and 50.00%, respectively. Notably, the overall metabolic intensity at 20°C was notably lower than at 25°C and 30°C (**Figure 4A**). Across all three temperatures, DSL01 exhibited high utilization rates for arbutin, L-arabinose, D-xylose, D-ribose, and L-lyxose, with arbutin showing the highest metabolic utilization rates at all temperatures. Regarding nitrogen sources, DSL01 metabolized 30, 51, and 46 nitrogen sources at 20°C, 25°C, and 30°C, respectively, with alanine-glycine complex being predominantly metabolized at 20°C, while L-tryptophan was primarily utilized at 25°C and 30°C (**Figure 4B**). Additionally, DSL01 metabolized 2, 15, and 11 phosphorus sources at 20°C, 25°C, and 30°C, respectively, with notable utilization rates for certain phosphorus sources, such as adenosine 3’,5’-cyclic phosphate. The strain also metabolized 7, 24, and 18 types of sulfur sources at 20°C, 25°C, and 30°C, respectively, with distinct utilization rates observed for sulfur sources like L-methionine sulfone and L-cysteine sulfenic acid (**Figure 4C**). Furthermore, analyses of DSL01’s metabolic phenotype under different stresses revealed variations in metabolic capacity under different ionic strengths and osmotic pressures. Notably, low concentrations of sodium chloride, potassium chloride, sodium formate, and sodium lactate sustained high metabolic capacity, while higher concentrations of ethylene glycol exhibited the strongest metabolic capacity. Conversely, exposure to 20~200 mmol/L sodium benzoate markedly reduced the metabolic ability of DSL01, particularly at 20°C (**Figure 4D**). These comprehensive findings provide deeper insights into the biological characteristics of *C. karstii* DSL01.

The results showed that the strongest pathogenicity of the pathogen was observed at 25°C, with 30°C being the second. Arbutin was identified as the most suitable carbon source, and its utilization varied at different temperatures. It was hypothesized that carbohydrate metabolism and pathogenicity were affected by temperature. Amino acids were the main substances in nitrogen source metabolism, with the greatest variety and highest intensity at 25°C. It was presumed that plant amino acids were utilized by pathogens, leading to the weakening of plant stress resistance. Regarding the phosphorus source, the highest utilization rate of adenosine 3’,5’-cyclic phosphate was detected at 25°C, which was speculated to regulate the synthesis of cellulase by modulating its absorption, thereby resulting in pathogenicity. Among the sulfur sources, L-cysteine sulfinic acid exhibited the highest utilization rate at 25°C, which was involved in the synthesis and affected the tricarboxylic acid cycle, consequently regulating the activity of pathogenic bacteria.

### 3.5 Evaluation of *in vitro* inhibition of DSL01 by different fungicides

The primary objective is to devise effective strategies for preventing and controlling Danshen leaf anthracnose. To this end, fifteen chemical agents with diverse modes of action were selected and initial screening was carried out via mycelial toxicity determination. The results revealed that at an agent concentration of 10 μg/ml, several agents exhibited inhibition rates exceeding 90%. Specifically, four agents—fluconazole, carbendazim, fluazinam, and pyraclostrobin—demonstrated notably high inhibitory effects. Notably, the strongest inhibitory effect was observed with the fluazinam, with an inhibition rate of 99.52%. Two other types, namely thiophanate-methyl and difenoconazole, displayed inhibition rates exceeding 80%. Lastly, triazolone, with an inhibition rate of 75.27%, was among the agents exhibiting inhibition rates exceeding 70% (**Supplementary Table S8**).

Subsequently, a detailed analysis of the inhibitory parameters of strain DSL01 by seven chemicals which exhibited inhibition rates above 70% was conducted. As expected, fluazinam exhibited the strongest inhibitory ability, with an *EC50* of 0.0725 μg/mL. This was followed by difenoconazole, fluconazole, triazolone, and carbendazim, with *EC50* values of 0.1093, 0.1293, 0.1635, and 0.2540 μg/mL, respectively. Conversely, triazolone and thiophanate-methyl exhibited poor inhibitory abilities, with *EC50* values of 2.3891 μg/mL and 1.1552 μg/mL, respectively (**Table 2**). Among the six selected botanical fungiticide, the most potent inhibitory effect on DSL01 mycelial growth was observed with 1% saccule aqueous emulsion, exhibiting an *EC50* of 4.8984 μg/mL. The *EC50* values for other treatments were as follows: 12.6141 μg/mL for 80% ethyl allicin cream, 19.9310 μg/mL for 5% carvavol solution, 23.6324 μg/mL for 2% sopranine water, and 28.8176 μg/mL for 6% oligosaccharide water; while the weakest inhibition was seen with a value of 52.5435 μg/mL for the use of a 20% eugenol aqueous emulsion (**Table 3**). Similarly, the effects of these chemicals on DSL01 spore germination were analyzed. It was observed that pyraclostrobin and fluazinam demonstrated the most effective inhibition of spore germination, with *EC50* values of 0.1175 μg/mL and 0.0378 μg/mL, respectively. Difenoconazole, fluconazole, and carbendazim followed, with *EC50* values of 27.2550, 24.8451, and 20.2605 μg/mL, respectively. Notably, two species exhibited *EC50* values exceeding 50 μg/mL: triazolone and thiophanate-methyl, with values of 191.5280 μg/mL and 84.3926 μg/mL, respectively (**Table 4**). In terms of botanical fungiticide, the most potent effect was observed with 80% ethicin milk EC50 at 0.5541μg/mL; while the *EC50* values for other treatments were 3.2656 μg/mL and 4.4560μg/mL, respectively. The less effective treatments included a 5% carvavol solution, eugenol aqueous emulsion, and a 6% oligosaccharide aqueous solution, with *EC50* values of 44.1769μg/mL, 80.2572μg/mL, and 62.8555μg/mL (**Table 5**). Based on these findings, fluazinam emerges as a standout candidate, displaying dual inhibitory effects on both mycelial growth and spore germination of DSL01. Therefore, it holds promise as an efficient agent for the prevention and control of anthracnose on Danshen leaves.

**Table 2:** Toxicity determination of seven chemical fungicides to DSL01 mycelium.

Fungicides	Toxic regression equation	*R*	*EC_50_* (μg/mL)	95% Confidence intervals
Pyraclostrobin	y=0.9278x+5.8243	0.9241	0.1293	0.0901~0.1857
Fluazinam	y=1.0463x+6.1926	0.9954	0.0725	0.0520~0.1010
Triazolone	y=0.9196x+4.6522	0.9925	2.3891	1.6491~3.4613
Difenoconazole	y=0.4897x+5.4709	0.9908	0.1093	0.0564~0.2117
Fluconazole	y=0.8021x+5.6309	0.9757	0.1635	0.1067~0.2505
Thiophanate-methyl	y=0.8451x+4.9471	0.9786	1.1552	0.7752~1.7213
Carbendazim	y=1.8733x+6.1150	0.977	0.254	0.2117~0.3047

**Table 3:** Toxicity determination of six botanical fungiticide to DSL01 mycelium.

Fungicides	Toxic regression equation	*R*	*EC_50_* (μg/mL)	95% Confidence intervals
5% carvacrol soluble solution	y=1.3829x+3.2029	0.8392	19.931	15.7871~25.1627
20% eugenol emulsion in water	y=1.6145x+2.2222	0.9604	52.5435	42.9424~64.2911
6% oligosaccharide aqueous solution	y=1.1643x+3.3006	0.9976	28.8176	21.8639~37.9829
1% osthole emulsion in water	y=1.0115x+4.3020	0.9963	4.8984	3.5016~6.8524
2% Matrine aqueous solution	y=0.6083x+4.1646	0.8891	23.6327	14.0719~38.6893
80% ethylicin EC	y=2.6200x+2.1157	0.8409	12.6141	11.0964~14.3392

**Table 4:** Toxicity determination of seven chemical fungicides to against spore germination of strain DSL01.

Fungicides	Toxic regression equation	*R*	*EC_50_* (μg/mL)	95% Confidence intervals
Pyraclostrobin	y=1.9413x+6.8057	0.9773	0.1175	0.0985~0.1401
Fluazinam	y=2.5618x+8.6428	0.8962	0.0378	0.0339~0.0422
Triazolone	y=2.8343x-1.4685	0.9705	191.528	170.9765~214.5497
Difenoconazole	y=1.9288x+2.2313	0.8868	27.255	23.7368~31.2947
Fluconazole	y=2.3433x+1.7305	0.9449	24.8451	21.8227~28.2862
Thiophanate-methyl	y=2.2128x+0.7374	0.9029	84.3926	74.9862~94.9789
Carbendazim	y=1.9848x+2.4065	0.9191	20.2605	17.4848~23.4769

**Table 5:** Toxicity determination of six botanical fungiticide to against spore germination of strain DSL01.

Fungicides	Toxic regression equation	*R*	*EC_50_* (μg/mL)	95% Confidence intervals
5% carvacrol soluble solution	y=2.4352x+0.9934	0.9024	44.1769	39.1868~49.8025
20% eugenol emulsion in water	y=2.5972x+0.0536	0.9492	80.2572	70.2822~91.6480
6% oligosaccharide aqueous solution	y=1.5832x+2.1529	0.8805	62.8555	52.5009~75.2522
1% osthole emulsion in water	y=1.7578x+4.0965	0.9864	3.2656	2.7863~3.8273
2% Matrine aqueous solution	y=1.9867x+3.7107	0.9924	4.456	3.8375~5.1742
80% ethylicin EC	y=2.1682x+5.5559	0.9932	0.5541	0.4827~0.6360

## 4 Discussion

A leaf disease affecting Danshen was observed in the cultivation area located in Hechuan District of Chongqing, China. Through morphological and molecular biological analyses, the pathogen DSL01 was identified as *C. karstii*. *C. karstii*, a member of the *C. boninense* complex, exhibits a broad geographical distribution and has the capacity to infect various plant species (Weir et al., 2012). In Brazil, this pathogen induces anthracnose in a diverse array of plants, including mayflower, passion fruit, mango, blueberry, jujube, and dragon fruit, significantly impacting Brazilian crop production (Sousa et al., 2021; Lima et al., 2013; Soares et al., 2021). Similarly, in China, there have been reports of plant anthracnose caused by *C. karstii*, such as anthracnose affecting rubber Tree, *Dalbergia odorifera*, as well as Litchi (Cai et al., 2016; Zhao et al., 2023, 2021). Through a comprehensive literature review, it is evident that the anthracnose disease caused by *C. karstii* on Danshen, as investigated in this study, represents the inaugural documented case both domestically and internationally.

Numerous studies have highlighted that anthrax caused by *Colletotrichum* tends to intensify during spring and summer, with the pathogen exhibiting high adaptability to neutral and alkaline environments (Alkan et al., 2008). Furthermore, suitable light conditions can stimulate the maturation of strain reproductive organs, thereby facilitating extensive sporulation (Mello et al., 2004). It is revealed by the findings of our study that the optimal temperature range for the growth of *C. karstii* mycelium is fallen between 10 and 35°C, with 25°C representing the most conducive temperature for growth, spore production, and pathogenicity. Notably, the pathogen demonstrates peak spore production under a 12-hour light and 12-hour darkness cycle, while mycelium growth thrives within a pH range of 5 to 9, consistent with previous research. This understanding of fundamental biological characteristics serves as a guide for devising effective strategies for the scientific prevention and control of anthracnose on Danshen leaves, based on environmental variations. Additionally, it underscores the importance of proactive medication measures before the optimal disease development period.

In addition to factors such as temperature and light, the metabolism of nutrients such as carbon and nitrogen sources also plays a crucial role in influencing the pathogenicity of pathogens (Solomon et al., 2003; Sun et al., 2024). To investigate the potential relationship between the pathogenicity of *C. karstii* and the metabolism of carbon and nitrogen sources, this study analyzed its utilization of different carbon and nitrogen sources at various pathogenic temperatures. It was observed that *C. karstii* exhibited the strongest pathogenicity at 25°C, coinciding with the highest efficiency in utilizing carbon and nitrogen sources, underscoring the significance of carbon and nitrogen source metabolism in *C. karstii* pathogenesis. Among the 190 different carbon source materials tested, arbutin demonstrated the highest metabolic utilization rate across all three temperatures, suggesting its suitability as a carbon source for the pathogen. Arbutin, a hexose aromatic carbohydrate, plays a role in cellular glycolysis, thus temperature variations may impact energy absorption by modulating carbohydrate metabolism, consequently influencing pathogenicity (An et al., 2023). Regarding nitrogen sources, L-tryptophan exhibited the highest metabolic intensity at 25°C. Tryptophan serves as a precursor substance for indoleacetic acid synthesis in plants, which has been linked to enhancing plant stress resistance (Wang et al., 2018). Upon invading plants, pathogens may exploit the amino acid composition within the plant, potentially compromising the plant’s ability to produce indoleacetic acid, thereby diminishing its resistance to pathogens and leading to disease development. Nonetheless, the intricate interplay between these carbon and nitrogen sources and *C. karstii* pathogenicity necessitates further exploration. Nevertheless, these findings emphasize the importance of judiciously managing carbon and nitrogen sources, along with other substances, during Danshen cultivation to prevent excessive application, which may exacerbate anthracnose occurrence on Danshen leaves.

While controversial, chemical control remains one of the most effective measures against anthracnose. Therefore, an *in vitro* screening for potentially effective fungicides against the pathogen was conducted and it was found that Fluazinam exhibited the strongest dual inhibitory effect on the mycelial growth and spore germination of *C. karstii*, aligning with previously reported optimal anthracnose control strategies (Rebello et al., 2022). These consistent results suggest that Fluazinam may possess high efficacy and a broad spectrum in preventing and treating anthracnose, indicating its potential for specifically managing other anthracnose diseases. Fluazinam, a dinitroaniline compound, operates through a unique mechanism that disrupts mitochondrial oxidative phosphorylation, thereby impeding fungal cell energy production (Vitoratos, 2014). It is speculated that spraying Fluazinam during the early stages of anthracnose could effectively eradicate a significant number of pathogenic fungal spores, inhibit population growth, and contribute to preventing anthracnose in Danshen. However, comprehensive field trials are warranted to validate these findings.

Among the 6 botanical fungicides, the 80% ethylicin emulsifiable concentrate and 1% osthole water emulsion have demonstrated robust efficacy in inhibiting mycelial growth and spore germination. Furthermore, Escoseed can be applied as a phytotoxin to mitigate plant infection and prevent pathogen reproduction within plants (Beier and Oertli, 1983). Ethylicin, developed as a synthetic broad-spectrum fungicide by the Shanghai Institute of Organic Chemistry, possesses a unique molecular structure -SS(=O)**_2_** that impacts the -SH-based material response in bacterial cells, thereby inhibiting normal cell metabolism (Hansen, 1972). While the indoor virulence test effect of these fungicides is relatively weaker than that of chemical fungicides, further validation through field efficacy tests is necessary to confirm their ultimate control effect.

In conclusion, our study successfully isolated *C. karstii* as the causal agent of anthracnose on Danshen leaves in Hechuan District, Chongqing, China. We thoroughly analyzed the biological characteristics of the *C. karstii* strain DSL01 and proposed scientific management recommendations for the prevention and control of Danshen leaf anthracnose using Fluazinam and 80% Ethylicin EC,1% Cnidium monnieri. Our findings offer valuable scientific insights and practical advice for effectively managing anthracnose in Danshen cultivation, thereby contributing to the sustainable production of this important medicinal herb.

## Data Availability

The raw data supporting the conclusions of this article will be made available by the authors, without undue reservation.

## References

[B1] Alkan N. Fluhr R. Sherman A. Prusky D. (2008). [amp]]lsquo;Role of ammonia secretion and pH modulation on pathogenicity of *Colletotrichum coccodes* on tomato fruit’. Mol. Plant-Microbe Interactions® 21, 1058–1066. doi: 10.1094/MPMI-21-8-1058 18616402

[B2] An N. Zhou S. Chen X. Wang J. Sun X. Shen X.. (2023). [amp]]lsquo;High-yield production of β-arbutin by identifying and eliminating byproducts formation’. Appl. Microbiol. Biotechnol. 107, 6193–6204. doi: 10.1007/s00253-023-12706-x 37597019

[B3] Beier R. C. Oertli E. H. (1983). Psoralen and other linear furocoumarins as phytoalexins in celery. Phytochemistry 22(11):2595–7. doi: 10.1016/0031-9422(83)80173-3

[B4] Boufleur T. R. Ciampi-Guillardi M. Tikami Ísis Rogério Flávia Thon M. R. Sukno S. A.. (2021). Soybean anthracnose caused by *Colletotrichum* species: Current status and future prospects. Mol. Plant Pathol. 22, 393–409. doi: 10.1111/mpp.13036 33609073 PMC7938629

[B5] Cai Z. Y. Liu Y. X. Shi Y. P. Mu H. J. Li. G. H. (2016). First report of leaf anthracnose caused by *Colletotrichum karstii* of rubber tree in China. Plant Dis. 100, 2528. doi: 10.1094/PDIS-04-16-0577-PDN

[B6] Cannon P. Damm U. Johnston P. Weir B. (2012). *Colletotrichum*–current status and future directions. Stud. mycol. 73, 181–213. doi: 10.3114/sim0014 23136460 PMC3458418

[B7] Cheng T. O. (2006). Danshen: What every cardiologist should know about this Chinese herbal drug. Int. J. Cardiol. 110, 411–412. doi: 10.1016/j.ijcard.2005.08.069 16730816

[B8] Damm U. Cannon P. F. Liu F. Barreto R. W. Guatimosim E. Crous P. W. (2013). The *Colletotrichum orbiculare* species complex: Important pathogens of field crops and weeds. Fungal Diversity 61, 29–59. doi: 10.1007/s13225-013-0255-4

[B9] Dean R. Van Kan J. A.L. Pretorius Z. A. Hammond-Kosack K. E. Di Pietro A. Spanu P. D.. (2012). The Top 10 fungal pathogens in molecular plant pathology. Mol. Plant Pathol. 13, 414–430. doi: 10.1111/j.1364-3703.2011.00783.x 22471698 PMC6638784

[B10] Fan G. Zou A. Wang X. Huang G. Tian J. Ma X.. (2022). Polymorphic microsatellite development, genetic diversity, population differentiation and sexual state of *Phytophthora capsici* on commercial peppers in three provinces of Southwest China. Appl. Environ. Microbiol. 88, e01611–e01622. doi: 10.1128/aem.01611-22 36354348 PMC9746301

[B11] Fang Y.-L. Xia L.-M. Wang P. Zhu L.-H. Ye J.-R. Huang L. (2018). The MAPKKK CgMck1 is required for cell wall integrity, appressorium development, and pathogenicity in *Colletotrichum gloeosporioides* . Genes 9, 543. doi: 10.3390/genes9110543 30413120 PMC6267176

[B12] Fernández-Herrera E. Rentería-Martínez MaríaE. Ramírez-Bustos I. I. Moreno-Salazar S. F. Ochoa-Meza Andrés Guillén-Sánchez D. (2020). *Colletotrichum karstii*: causal agent of anthracnose of *Dendrobium nobile* in Mexico. Can. J. Plant Pathol. 42, 514–519. doi: 10.1080/07060661.2020.1731711

[B13] Garcia-Lopez M. T. Gordon A. Raya M. C. Diez C. M. Moral J. (2020). First report of *Colletotrichum karstii* causing fruit anthracnose of *Carissa grandiflora* in Spain. Plant Dis. 105, 492–492. doi: 10.1094/PDIS-07-20-1581-PDN

[B14] Glass N. L. Donaldson G. C. (1995). Development of primer sets designed for use with the PCR to amplify conserved genes from filamentous ascomycetes. Appl. Environ. Microbiol. 61, 1323–1330. doi: 10.1128/aem.61.4.1323-1330.1995 7747954 PMC167388

[B15] Han S. Li Na Zhang R. Hu X. Zhang W. Zhang Bo. (2023). Study on signal transmission mechanism of arbuscular mycorrhizal hyphal network against root rot of *Salvia miltiorrhiza* . Sci. Rep. 13, 16936. doi: 10.1038/s41598-023-43278-5 37805532 PMC10560300

[B16] Hansen J. C. (1972). The effect of some sulphur and mercury containing fungicides on bacteria. Chemosphere 1, 159–162. doi: 10.1016/0045-6535(72)90020-3

[B17] Hong S. G. Cramer R. A. Lawrence C. B. Pryor B. M. (2005). Alt a 1 allergen homologs from *Alternaria* and related taxa: analysis of phylogenetic content and secondary structure. Fungal Genet. Biol. 42, 119–129. doi: 10.1016/j.fgb.2004.10.009 15670710

[B18] Jia F. Chen M. Liu C. Chen S. Liu W. Huang K.. (2024). *Streptomyces rapamycinicus* HCD1–10: An effective biocontrol actinomycetes against postharvest Chinese flat peach brown rot caused by *Monilinia fructicola* . Scientia Hortic. 327, 112836. doi: 10.1016/j.scienta.2023.112836

[B19] Jin Y. Fei W. Yi W. Suxia G. Chuantao Lu Yuxia L.. (2020). First report of *Fusarium proliferatum* causing root rot disease in *Salvia miltiorrhiza* in China. Plant Dis. 105, 1210–1210. doi: 10.1094/PDIS-09-20-1908-PDN

[B20] Khatri B. Fielder M. Jones G. Newell W. Abu-Oun M. Wheeler P. R. (2013). High throughput phenotypic analysis of *Mycobacterium tuberculosis* and *Mycobacterium bovis* strains’ metabolism using BIOLOG phenotype microarrays. PloS One 8, e52673. doi: 10.1371/journal.pone.0052673 23326347 PMC3542357

[B21] Kum Ka Y. Kirchhof R. Luick R. Heinrich M. (2021). Danshen (*Salvia miltiorrhiza*) on the global market: What are the implications for products’ quality? Front. Pharmacol. 12. doi: 10.3389/fphar.2021.621169 PMC8107819 33981218

[B22] Lima N. B. Marques M. W. Michereff S. J. Morais M. A. Barbosa M. A. G. Câmara. M. P. S. (2013). First report of mango anthracnose caused by *Colletotrichum karstii* in Brazil. Plant Dis. 97, 1248–1248. doi: 10.1094/PDIS-01-13-0002-PDN 30722427

[B23] Lu P. K. Pan H. R. Lin X. R. (2018). First report of *Corynespora cassiicola* causing leaf spot on *Salvia miltiorrhiza* in Taiwan. Plant Dis. 103, 769. doi: 10.1094/PDIS-07-18-1155-PDN

[B24] Mayorquin J. S. Nouri M. T. Peacock B. B. Trouillas F. P. Douhan G. W. Kallsen C.. (2019). Identification, pathogenicity, and spore trapping of *Colletotrichum karstii* associated with twig and shoot dieback in California. Plant Dis. 103, 1464–1473. doi: 10.1094/PDIS-08-18-1425-RE 30998450

[B25] Mello A. MaChado A. Bedendo I. (2004). Development of Colletotrichum gloeosporioides isolated from green pepper in different culture media, temperatures, and light regimes. Scientia Agricola 61, 542–544. doi: 10.1590/S0103-90162004000500013

[B26] Nascimento M. B. Bellé C. Azambuja R. M. Maich S. L. P. Neves C. G. Souza-Junior I. T.. (2019). First report of *Colletotrichum karstii* causing anthracnose spot on pitaya (*Hylocereus undatus*) in Brazil. Plant Dis. 103, 2137. doi: 10.1094/PDIS-02-19-0400-PDN

[B27] Pan S. Wang L. Zhang R. Wei P. Y. Yang Y. W. Peng D. L.. (2022). First report of *Meloidogyne hapla* infecting *Salvia miltiorrhiza* in Shaanxi, China. Plant Dis. 107, 585. doi: 10.1094/PDIS-01-22-0088-PDN

[B28] Pu C. Ge Y. Yang G. Zheng H. Guan W. Chao Z.. (2022). Arbuscular mycorrhizal fungi enhance disease resistance of *Salvia miltiorrhiza* to *Fusarium* wilt. Front. Plant Sci. 13. doi: 10.3389/fpls.2022.975558 PMC9753693 36531366

[B29] Qian J. Song J. Gao H. Zhu Y. Xu J. Pang X.. (2013). The complete chloroplast genome sequence of the medicinal plant *Salvia miltiorrhiza* . PloS One 8, e57607. doi: 10.1371/journal.pone.0057607 23460883 PMC3584094

[B30] Rebello C. S. Baggio J. S. Forcelini B. B. Peres N. A. (2022). Sensitivity of *Colletotrichum acutatum* species complex from strawberry to fungicide alternatives to quinone-outside inhibitors. Plant Dis. 106, 2053–2059. doi: 10.1094/PDIS-09-21-1934-RE 35285270

[B31] Sa R. He S. Han D. Liu M. Yu Y. Shang R.. (2022). Isolation and identification of a new biocontrol bacteria against *Salvia miltiorrhiza* root rot and optimization of culture conditions for antifungal substance production using response surface methodology. BMC Microbiol. 22, 231. doi: 10.1186/s12866-022-02628-5 36180825 PMC9524000

[B32] Schoch C. L. Seifert K. A. Huhndorf S. Robert V. Spouge J. L. Levesque C.André. (2012). Nuclear ribosomal internal transcribed spacer (ITS) region as a universal DNA barcode marker for *Fungi* . Proc. Natl. Acad. Sci. 109, 6241–6246. doi: 10.1073/pnas.1117018109 22454494 PMC3341068

[B33] Soares V. F. Velho A. C. Carachenski A. Astolfi P. Stadnik. M. J. (2021). First report of *Colletotrichum karstii* causing anthracnose on strawberry in Brazil. Plant Dis. 105, 3295. doi: 10.1094/PDIS-03-21-0518-PDN

[B34] Solomon P. S. Tan K.-C. Oliver R. P. (2003). The nutrient supply of pathogenic fungi; a fertile field for study. Mol. Plant Pathol. 4, 203–210. doi: 10.1046/j.1364-3703.2003.00161.x 20569380

[B35] Song L. Zhong R. Guan Z. Huang L. Wang G. Yang Z.. (2024). Molecular characterization of the first partitivirus from a causal agent of *Salvia miltiorrhiza* dry rot. J. Fungi 10, 179. doi: 10.3390/jof10030179 PMC10971513 38535188

[B36] Sousa E. S. de Sousa Carvalho G. Barguil B. M. da Silva Matos K. Beserra JoséE. A. (2021). First report of anthracnose on *Spigelia anthelmia* caused by *Colletotrichum karstii* and *Colletotrichum siamense* in Brazil. J. Plant Dis. Prot. 128, 875–880. doi: 10.1007/s41348-021-00449-8

[B37] Sun M. Dai P. Cao Z. Dong J. (2024). Purine metabolism in plant pathogenic fungi. Front. Microbiol. 15. doi: 10.3389/fmicb.2024.1352354 PMC10879430 38384269

[B38] Talhinhas P. Baroncelli R. (2023). Hosts of *colletotrichum* . mycosphere 14, 158–261. doi: 10.5943/mycosphere/14/si2/4

[B39] Than P. P. Jeewon R. Hyde K. D. Pongsupasamit S. Mongkolporn O. Taylor. P. W. J. (2008). Characterization and pathogenicity of *Colletotrichum* species associated with anthracnose on chilli (*Capsicum* spp.) in Thailand. Plant Pathol. 57, 562–572. doi: 10.1111/j.1365-3059.2007.01782.x

[B40] Vitoratos A. G. (2014). Mode of action and genetic analysis of resistance to Fluazinam in *Ustilago maydis* . J. Phytopathol. 162, 737–746. doi: 10.1111/jph.2014.162.issue-11-12

[B41] Wang W. Liang X. Zhang R. Gleason M. L. Sun G. (2017). Liquid shake culture overcomes solid plate culture in inducing conidial production of *Colletotrichum* isolates. Australas. Plant Pathol. 46, 285–287. doi: 10.1007/s13313-017-0490-3

[B42] Wang G. Ma C.-J. Luo Yi Zhou S.-S. Zhou Y. Ma X.-L.. (2018). Proteome and transcriptome reveal involvement of heat shock proteins and indoleacetic acid metabolism process in *Lentinula Edodes* Thermotolerance. Cell. Physiol. Biochem. 50, 1617–1637. doi: 10.1159/000494784 30384356

[B43] Weir B. Johnston P. Damm U. (2012). The *Colletotrichum gloeosporioides* species complex. Stud. mycol. 73, 115–180. doi: 10.3114/sim0011 23136459 PMC3458417

[B44] Wen Yi Chen K. Cui J. Wang T. Zhang H. Zheng F.. (2022). First report of the root-knot nematode *Meloidogyne incognita* on *Salvia miltiorrhiza* in Henan Province, China. Plant Dis. 107, 969. doi: 10.1094/PDIS-05-22-0997-PDN

[B45] Wu Z. M. Chen Y. Xie X. L. Wen C. X. Chen J. F. Zhou. M. G. (2008). Note: Occurrence and distribution of *Cucumber mosaic virus* infecting danshen (*Salvia miltiorrhiza*) in China. Phytoparasitica 36, 217–219. doi: 10.1007/BF02980766

[B46] Xing Z. Bi G. Li T. Zhang Q. Knight P. R. (2023a). Nitrogen fertilization improves growth and bioactive compound content for *Salvia miltiorrhiza* Bunge. Horticulturae 9, 254. doi: 10.3390/horticulturae9020254

[B47] Xing Z. Bi G. Li T. Zhang Q. Knight P. R. (2023b). Plant growth and the contents of major bioactive compounds of *Salvia miltiorrhiza* Bunge grown in Mississippi, United States. Horticulturae 9, 310. doi: 10.3390/horticulturae9030310

[B48] Yang Li Miao Z.-Q. Yang G. Shao A.-J. Huang qi Shen Ye. (2013). Research wilt disease of *Salvia miltiorrhiza* and its pathogen. China J. Chin. Materia Med. 38, 4040–4043. 24791484

[B49] Youlian Y. Cai L. Yu Z. Liu Z. Hyde K. (2011). *Colletotrichum* species on *orchidaceae* in Southwest China. Cryptogamie Mycologie 32, 229–253. doi: 10.7872/crym.v32.iss3.2011.229

[B50] Zhang L. Tao S. Zhang Y. Yang Y. Peng F. Liao H.. (2024). Study on the effect of compound cultivation on the growth feature and active ingredients content of *Salvia miltiorrhiza* . Front. Plant Sci. 14. doi: 10.3389/fpls.2023.1238896 PMC10854009 38343765

[B51] Zhao J. Sun J. Li M. Gong D. Yang J. He Y.. (2023). First report of anthracnose caused by *Colletotrichum karstii* on *Dalbergia odorifera* in China. Plant Dis. 108, 222. doi: 10.1094/PDIS-08-23-1592-PDN

[B52] Zhao J. Yu Z. Wang Y. Li Q. Tang L. Guo T.. (2021). Litchi anthracnose caused by *Colletotrichum karstii* in Guangxi, China. Plant Dis. 105, 3295. doi: 10.1094/PDIS-01-21-0196-PDN

