# Supplementary material for: Identification and management of a novel Danshen leaf anthracnose caused by Colletotrichum karstii in Salvia miltiorrhiza Bunge in China
Source: Front Plant Sci. 2025 Feb 4;16:1526038. doi: 10.3389/fpls.2025.1526038 (PMC11832477; doi:10.3389/fpls.2025.1526038)
Supplement: Supplementary file 1 [file DataSheet1.pdf]

Appendix A. Supplementary data

Fig. S1: The phylogenetic tree of strain DSM based on *ITS*, *LSU* and *RPB2* gene sequences. Specimens using the maximum likelihood method (1000 bootstrap iterations).

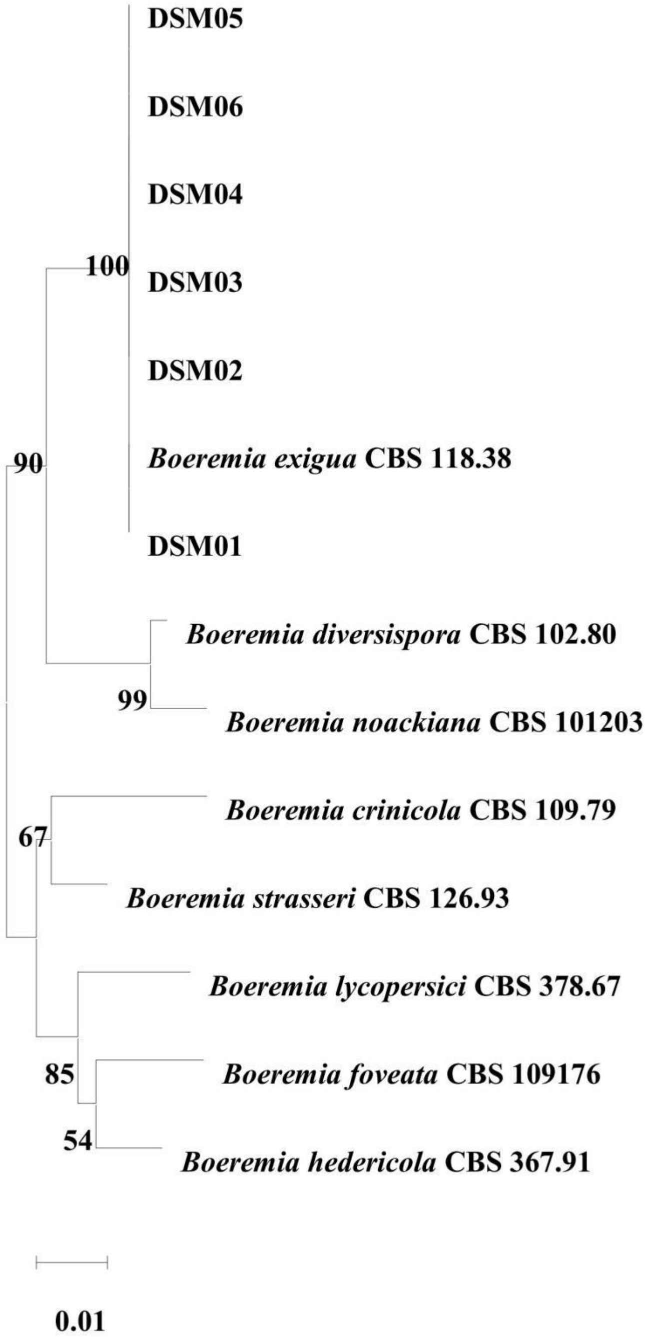

Fig. S2: The phylogenetic tree of strain DSN based on *RPB2*, *TEF1* and *Alt al* gene sequences. Specimens using the maximum likelihood method (1000 bootstrap iterations).

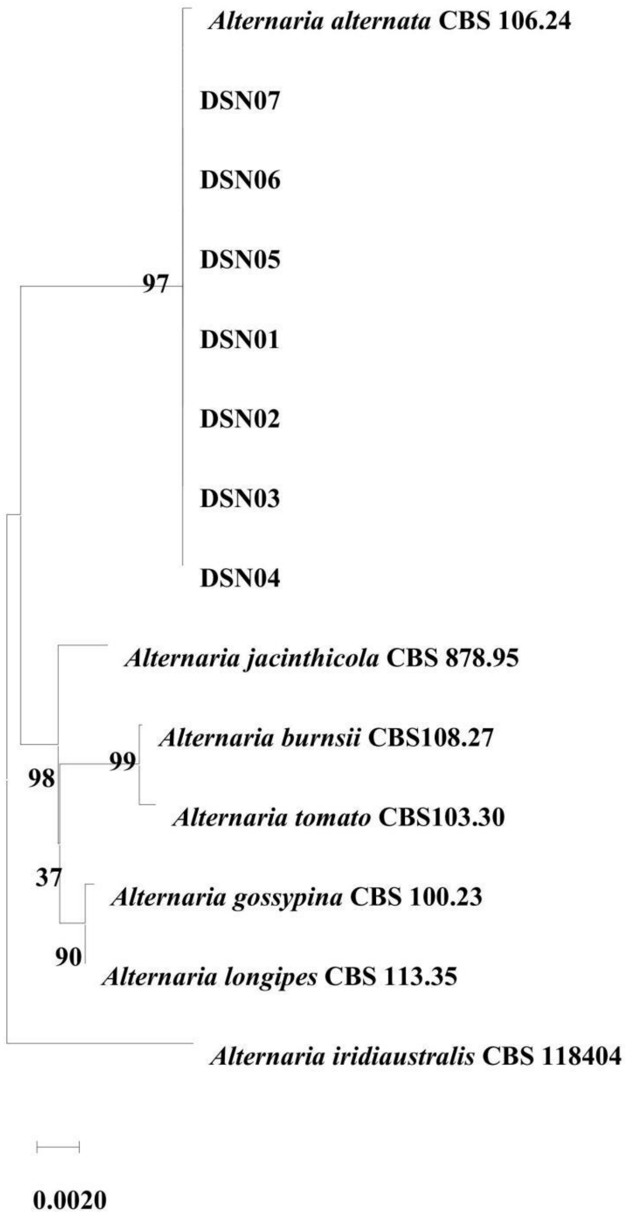

Fig. S3: Pathogenicity testing for strain DSM01

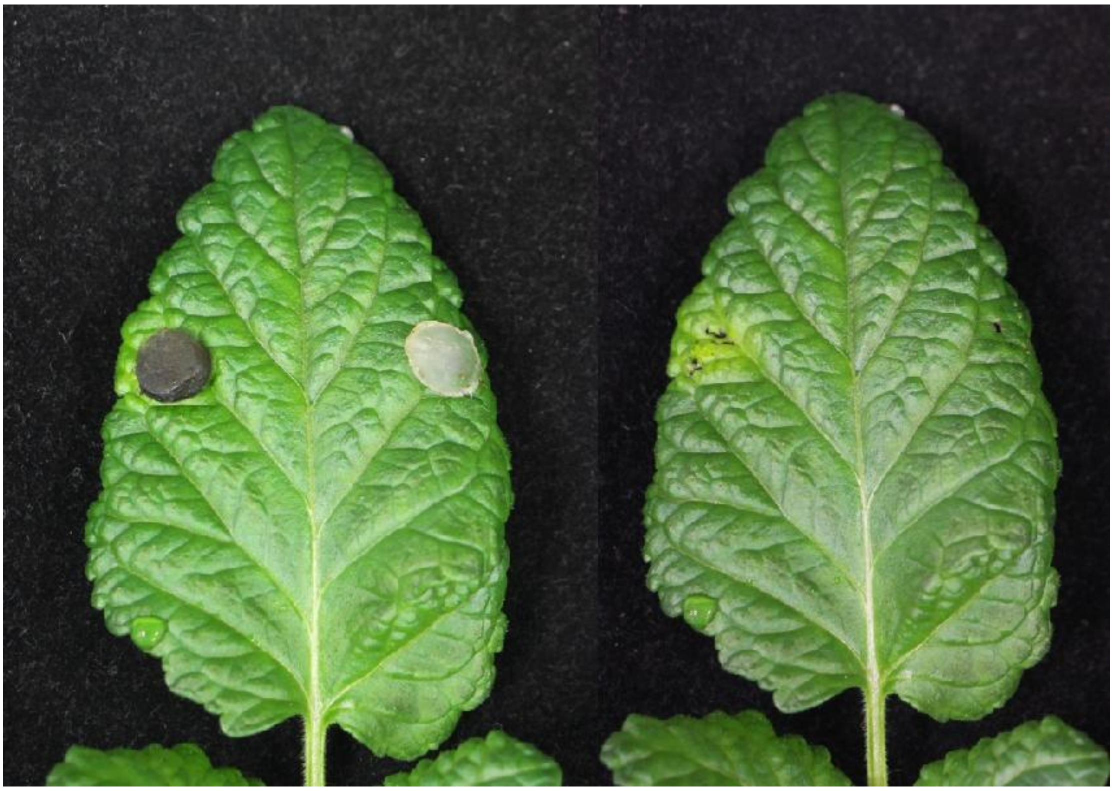

Fig. S4 Pathogenicity testing for strain DSN01.

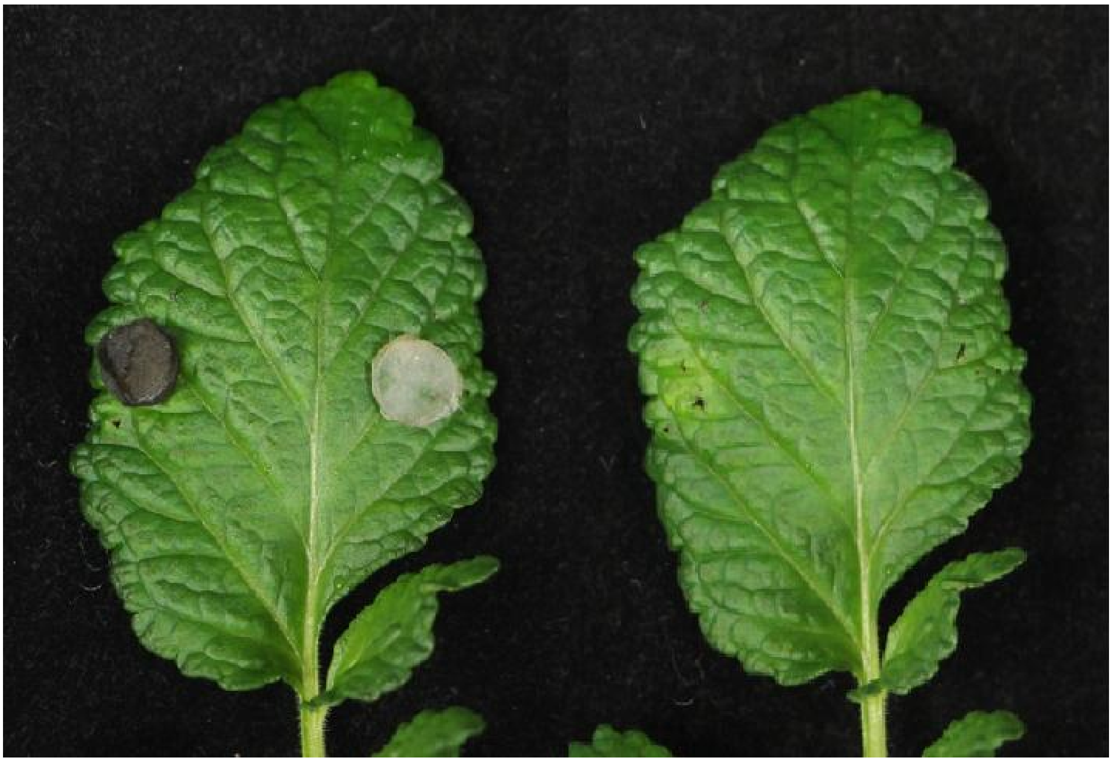

Table S1: Mediums used in the study.

Name of media	Ingredient
Potato Glucose Agar (PDA) Medium	Potato 200 g, glucose 20 g, agar 20 g, distilled water 1000 mL.
Water agar (WA) medium	Agar 20 g, distilled water 1000 mL.
Complete (CM) medium	Yeast extract 6 g, casein hydrolysate 6 g, sucrose 10 g, distilled water 1000 mL. Solid medium plus agar 20g/L

Note: The above-mentioned culture medium was autoclaved at 121℃ for 30 minutes.

Table S2: Primers used in the study.

Locus/target gene	Primer	Primer DNA Sequence (5′–3′)	Annealing temperature
Internal Transcribed Spacers (ITS)	ITS1	TCCGTAGGTGAACCTGCGG	55°C
	ITS4	TCCTCCGCTTATTGATATGC	
Allergen Alt a1 (Alt al)	*Alt*-F	ATGCAGTTCACCACCATCGC	59°C
	Alt-R	ACGAGGGTGAYGTAGGCGTC	
Histone 3 (His3)	*HIS*3-F	ACTAAGCAGACCGAAAGCAGG	66°C
	*HIS*3-R	CCGGGCGAGCTGGATCTCCTT	
Translation elongation factor 1-α gene (tef1)	EF1-728F	CATCGAGAAGTTCGAGAAGG	55°C
	EF1-986R	CATCGAGAAGTTCGAGAAGG	
Ribosomal Large Subunit (LSU)	LROR	GTACCCGCTGAACTTAAGC	53°C
	LR7	TACTACCACCAAGATCT	
β-tubulin (TUB)	Bt2a	GGTAACCAAATCGGTGCTGCTTTC	55°C
	Bt2b	ACCCTCAGTGTAGTGACCCTTGGC	
The second lar-gest RNA polymerase subunit (RPB2)	RPB2-5F2	GCCTTCTTCTG(G/A)TC(T/A)CCCC	64°C
	RPB2-7cR	CCCATR(A/G)GCTTGY(C/T)TTR(A/G)CCCAT	

Table S3:Related strain information and its genetic accession number

Species	Isolate	GenBank accessions						
		ITS	TUB	*His*3	RPB2	*Alt* a1	TEF1	LSU
Colletotrichum.abscissum	COAD1877	KP843126	KP843135	KP843141	—	—	—	—
C. acerbum	CBS 128530	JQ948459	JQ950110	JQ949450	—	—	—	—
C. acutatum	CBS 112996	JQ005776	JQ005860	JQ005818	—	—	—	—
C. beeveri	CBS 128527	JQ005171	JQ005605	JQ005432	—	—	—	—
C. boninense	CBS 123755	JQ005153	JQ005588	JQ005414	—	—	—	—
C. brisbanense	CBS 292.67	JQ948291	JQ949942	JQ949282	—	—	—	—
C. cairnsense	BRIP 63642	KU923672	KU923688	KU923722	—	—	—	—
C. cereale	CBS 129663	JQ005774	JQ005858	JQ005816	—	—	—	—
C. constrictum	CBS 128504	JQ005238	JQ005672	JQ005499	—	—	—	—
C. curcunae	IMI 288937	GU227893	GU228187	GU228089	—	—	—	—
C. dacrycarpi	CBS 130241	JQ005236	JQ005670	JQ005497	—	—	—	—
C. fioriniae	CBS 128517	JQ948292	JQ949943	JQ949283	—	—	—	—
C. gloeosporioides	CBS 112999	JQ005152	JQ005239	JQ005413	—	—	—	—
C. godetiae	CBS 133.44	JQ948402	JQ950053	JQ949393	—	—	—	—

C. hippeastri	CBS 125376	JQ005231	JQ005665	JQ005492	—	—	—	—
C. indonesiense	CBS 127551	JQ948288	JQ949939	JQ949279	—	—	—	—
C. karstii	CBS 127597	JQ005204	JQ005638	JQ005465	—	—	—	—
C. lacticiphilum	CBS 112989	JQ948289	JQ949940	JQ949280	—	—	—	—
C. lupini	CBS 109225	JQ948155	JQ949806	JQ949146	—	—	—	—
C. melonis	CBS 159.84	JQ948194	JQ949845	JQ949185	—	—	—	—
C. nymphaeae	CBS 515.78	JQ948197	JQ949848	JQ949188	—	—	—	—
C. oncidii	CBS 129828	JQ005169	JQ005603	JQ005430	—	—	—	—
C. pyricola	CBS 128531	JQ948445	JQ950096	JQ949436	—	—	—	—
C. sloanei	IMI 364297	JQ948287	JQ949938	JQ949278	—	—	—	—
C. tamarilloi	CBS 129814	JQ948184	JQ949835	JQ949175	—	—	—	—
Alternaria. alternata	CBS 106.24	—	—	—	KP124766	KP123847	KP125073	—
A. burnsii	CBS 108.27	—	—	—	KC584468	KP123850	KC584727	—
A. iridiaustralis	CBS 118404	—	—	—	KP124904	KP123980	KP125213	—
A. gossypina	CBS 100.23	—	—	—	KP124899	KP123977	KP125208	—

A. longipes	CBS 113.35	—	—	—	KP124910	KP123986	KP125219	—
A. tomato	CBS 103.30	—	—	—	KP124915	KP123991	KP125224	—
A. jacinthicola	CBS 878.95	—	—	—	KP124907	KP123983	KP125216	—
Boeremia. exigua	CBS118.38	KT389489	—	—	KT389582	—	—	KT389706
B. crinicola	CBS109.79	GU237737	—	—	KT389563	—	—	GU237927
B. diversispora	CBS102.80	GU237725	—	—	KT389565	—	—	GU237930
B. foveata	CBS 109176	GU237742	—	—	KT389578	—	—	GU237946
B. hedericola	CBS 367.91	GU237842	—	—	KT389579	—	—	GU237949
B. lycopersici	CBS 378.67	GU237848	—	—	KT389580	—	—	GU237950
B. noackiana	CBS 101203	GU237720	—	—	KT389581	—	—	GU237953
B. strasseri	CBS 126.93	GU237773	—	—	KT389584	—	—	GU237956

Table S4:Chemical fungicides information.

**Mode of action**	**Chemical structure**	**Active principle**	**Percent**	**Manufacture**
Inhibiting respiration	Methoxy acrylate	Azoxystrobin	98.25%	Hubei Kangbaotai Fine Chemical Co., Ltd.
	The methoxy acrylic acid class	Pyraclostrobin	98%	Tianjin Hanbang Plant Protective Agent Co., Ltd.
	Dinitroanilines	Fluazinam	98%	Tianjin Hanbang Plant Protective Agent Co., Ltd.
	Formamides	Thifluzamide	98%	Tianjin Hanbang Plant Protective Agent Co., Ltd.
Multisite joint activity	Thiocarbamate	Mancozeb	80%	Hubei Kangbaotai Fine Chemical Co., Ltd.
Suppress signal conversion	Phenylpyrroles	Fludioxonil	98%	Tianjin Hanbang Plant Protective Agent Co., Ltd.
Inhibition of sterol biosynthesis in membrane	Triazoles	Triazolone	95%	Hubei Kangbaotai Fine Chemical Co., Ltd.
		Difenoconazole	95%	Hubei Kangbaotai Fine Chemical Co., Ltd.
	Imidazoles	Fluconazole	97%	Tianjin Hanbang Plant Protective Agent Co., Ltd.

Inhibition of mitosis and cell division	Thiocarbamates	Thiophanate-methyl	97.58%	Tianjin Hanbang Plant Protective Agent Co., Ltd.
	Benzimidazoles	Carbendazim	97.30%	Hubei Kangbaotai Fine Chemical Co., Ltd.
Inhibition of nucleic acid synthesis	Isoxazoles	Evil Grimm	97%	Tianjin Hanbang Plant Protective Agent Co., Ltd.
	Dicarboxyimides	Iprodione	95%	Hubei Kangbaotai Fine Chemical Co., Ltd.
Inhibition of membranou s and membrane synthesis	Organic phosphorus	Methyl pyrrolidine phosphate	97%	Tianjin Hanbang Plant Protective Agent Co., Ltd.
Inhibit the synthesis of melanin in cell wall	Triazole benzothiazole	Tricyclazole	97%	Tianjin Hanbang Plant Protective Agent Co., Ltd.

Table S5:Botanical fungiticide information.

Mode of action	Active principle	Percent	Dosage form	Manufacture
Inhibition of spore germination and mycelial growth	Carvacrol	5%	Soluble agent	Jiangsu Jianpai Agrochemical Co., Ltd.
	Matrine	2%	Water agent	Hebei Ruibaode Biochemistry Co., Ltd.
Inhibit the formation of cell wall	Eugenol	20%	Water emulsion	Jiangsu Jianpai Agrochemical Co., Ltd.
Induced plant resistance	Oligosaccharides	6%	Water agent	Hainan Zhengye Zhongnong High-tech Co., Ltd.
It inhibited the absorption of Ca^{+} by bacteria.	Osthole	1%	Water emulsion	Inner Mongolia Qingyuan Bao Biotechnology Co., Ltd.
Inhibit the normal metabolism of bacteria	Ethylicin	80%	Emulsifiable oil	Shandong Dezhou Xianglong Biochemical Co., Ltd.

Table S6: Concentration of each fungicide for *in vitro* toxicity test of mycelium.

**Fungicides**	**Concentrations of effective components (μg/mL)**				
Pyraclostrobin	0.02	0.08	0.32	1.28	5.12
Fluazinam	0.01	0.04	0.16	0.64	2.56
Triazolone	0.08	0.32	1.28	5.12	20.48
Difenoconazole	0.001	0.01	0.1	1	10
Fluconazole	0.08	0.32	1.28	5.12	20.48
Thiophanate-methyl	0.04	0.16	0.64	2.56	10.24
Carbendazim	0.04	0.08	0.16	0.32	0.64
5 % carvacrol soluble solution	5	10	20	40	80
20 % eugenol emulsion in water	10	20	40	80	160
6 % oligosaccharide aqueous solution	20	40	80	160	320
1 % osthole emulsion in water	0.625	2.5	10	40	160
2 % Matrine aqueous solution	5	20	80	320	640
80 % ethylicin EC	1	10	20	30	40

Table S7: Concentration of each fungicide for *in vitro* toxicity test of spore germination

**Fungicides**	**Concentrations of effective components (μg/mL)**				
Pyraclostrobin	0.1	0.2	0.4	0.8	1.6
Fluazinam	0.02	0.04	0.06	0.08	0.1
Triazolone	50	100	200	400	800
Difenoconazole	10	20	30	40	50
Fluconazole	5	10	20	40	80
Thiophanate-methyl	40	60	80	100	120
Carbendazim	4	8	16	32	64
5 % carvacrol soluble solution	10	30	50	70	90
20 % eugenol emulsion in water	10	20	40	80	160
6 % oligosaccharide aqueous solution	10	20	40	80	160
1 % osthole emulsion in water	1	2	4	8	16
2 % Matrine aqueous solution	2	4	8	16	32
80 % ethylicin EC	0.2	0.4	0.8	1.6	3.2

Table S8: Inhibition of hyphae growth of 15 chemical fungicides at a concentration of 10 μg/mL of DSL01

**Fungicides**	**Colony diameter (cm)**	**Inhibition rate of colony growth (%)**
Pyraclostrobin	0.85±0.03	96.43 ab
Fluazinam	0.63±0.02	99.52 a
Mancozeb	5.67±0.15	27.72 f
Fludioxonil	4.37±0.04	46.27 e
Azoxystrobin	4.42±0.02	45.55 e
Carbendazim	0.75±0.03	97.86 ab
Triazolone	2.33±0.03	75.27 d
Difenoconazole	1.35±0.05	89.30 c
Fluconazole	1.03±0.02	93.82 b
Thiophanate-methyl	1.63±0.16	85.26 c
Thifluzamide	6.68±0.19	13.22 h
Evil Grimm	6.53±0.03	15.36 gh
Iprodione	7.15±0.13	6.56 i
Methyl pyrrolidine phosphate	6.45±0.15	16.55 gh
Tricyclazole	6.33±0.16	18.21 g
CK	7.61±0.11	——
